# Structural and Magnetoelectrical Properties of MFe_2_O_4_ (M = Co, Ni, Cu, Mg, and Zn) Ferrospinels Synthesized via an Egg-White Biotemplate

**DOI:** 10.1021/acsomega.1c02858

**Published:** 2021-08-19

**Authors:** Mohamed A. Gabal, Dina F. Katowah, Mahmoud A. Hussein, A. A. Al-Juaid, Ayman Awad, A. M. Abdel-Daiem, Abdu Saeed, Mahmoud M. Hessien, Abdullah M. Asiri

**Affiliations:** † Chemistry Department, Faculty of Science, King Abdulaziz University, Jeddah 21589, Saudi Arabia; ‡ Chemistry Department, Faculty of Science, Benha University, Benha 30311, Egypt; § Department of Chemistry, Faculty of Applied Science, Umm Al-Qura University, Makkah 21421, Saudi Arabia; ∥ Polymer Chemistry Laboratory, Chemistry Department, Faculty of Science, Assiut University, Assiut 71516, Egypt; ⊥ Chemistry Department, Faculty of Science, University of Jeddah, Jeddah 21959, Saudi Arabia; # Chemistry Department, Faculty of Science, Benha University, Benha 30311, Egypt; ¶ Physics Department, Faculty of Science, King Abdulaziz University, Jeddah 21589, Saudi Arabia; ∇ Physics Department, Faculty of Science, Zagazig University, Zagazig 44519, Egypt; ○ Department of Physics, Thamar University, Thamar 87246, Yemen; ⧫ Department of Chemistry, College of Science, Taif University, Taif 21974, Saudi Arabia; †† Center of Excellence for Advanced Materials Research, King Abdul Aziz University, Jeddah 21589, Saudi Arabia

## Abstract

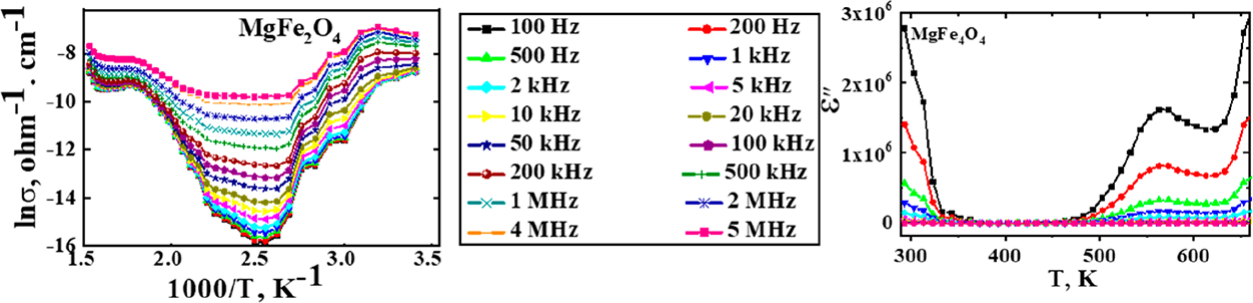

Nanocrystalline metal ferrites (MFe_2_O_4_, M = Co, Ni, Cu, Mg, and Zn) were successfully synthesized via autocombustion synthesis using egg white. X-ray diffraction (XRD) measurements revealed the crystallization of the entire ferrites either in the tetragonal structure, such as in the case of CuFe_2_O_4_, or cubic spinels such as in other studied ferrites. The Fourier transform infrared spectral study revealed the characteristic vibration bands of ferrites. Compared to other synthesis methods, the observed variation in the obtained structural parameters could be due to the different cation distribution of the prepared ferrites. In agreement with XRD measurements, the transmission electron microscopy images showed agglomerated particles with cubic morphology for all ferrites. On the other hand, CuFe_2_O_4_ showed tetragonal morphology. The magnetization values were found to vary with the type of the metal ion, and CoFe_2_O_4_ showed the highest one (42.8 emu/g). Generally, the lower magnetization values obtained than those reported in the literature for all studied ferrites could be attributed to the smaller particle sizes or the cation redistribution. The obtained coercivity values are observed to be higher than their related values in the literature, exhibiting the impact of the present synthesis route. Ac-conductivity as a function of temperature and frequency indicated semiconducting properties with the observed change in the conduction mechanism by increasing the temperature. The obtained low dielectric constant values could suggest using the entire ferrites in high-frequency applications such as microwave devices.

## 1 Introduction

Soft ferrites with the formula MFe_2_O_4_ (M = transition metals) are important members of ceramic oxides. They have been extensively investigated due to their acceptable magnetoelectrical properties, facilitate their enormous applications in many electronic devices.^1^ The tailored properties arise from their semiconducting behaviors at low temperatures, and their ferromagnetic properties under applied magnetic fields enriched their applications in magnetic data storage, high-frequency telecommunication, magnetic recording, ceramics tile industry, thin-film technology, photocatalysts, and microwave sensors.^2−8^

Conventionally, these spinel ferrites can be synthesized using the solid-state ceramic method,^9^ but the production of agglomerated irregularly shaped large particles diminishes in this way. On the other hand, the production of nanoscale particles is of great importance owing to their electrical, magnetic, and optical properties that are strongly dependent on the shape and size.^10^ Therefore, the careful control of the synthesis process remains challenging, justifying any effort to establish an economical, simple, and environmentally friendly route for synthesizing these size-tuned nanoparticles. Recently, techniques such as sol–gel offered an opportunity to prepare many nanoparticle systems with many desired properties; however, the use of toxic solvents, harsh conditions, and expensive materials limited their use.^11^ Hence, there will be more need to develop a convenient, economical, and nontoxic route to prepare such nanoparticles.

Recently, a relatively new, simple, economical, and environmentally friendly solution combustion method was motivated.^12−15^ In this method, a solution containing stoichiometric ratios of entire metal nitrates was mixed with an egg-white (ovalbumin) fuel and heated under vigorous stirring until autocombustion occurs. The produced nanocrystalline ferrite powders, in this case, could be used without any further heat treatments. The proper autocombustion mechanism for ferrite synthesis was previously described by Gabal et al.^15^

The prominent members of the spinel ferrites, MFe_2_O_4_ (M = Ni, Co, Mg, Cu, and Zn), have already received many researchers’ attention, aiming at their facile production, structure characterization, and investigation their different promising properties.^10,16−24^ The main aim here is to prepare a family of single spinel-phase nanocrystalline ferrites (MFe_2_O_4_) with M = Ni, Co, Mg, Cu, and Zn utilizing a fresh egg-white binder as a natural-cum-gelling material to modify their properties and crystal size. This convenient route could be utilized for the mass production of entire ferrites under mild conditions without any organic solvents. In this way, a simple, economical, low-reaction-time, and environment friendly method could be achieved.^25,26^

The ferrites’ formation, structure, and morphological properties were analyzed via X-ray diffraction (XRD), Fourier transform infrared (FT-IR), and transmission electron microscopy (TEM) techniques. The obtained magnetic characteristics were studied using vibrating sample magnetometry (VSM) measurements. Electrical properties, viz. conductivity and dielectric constant, were measured along with temperature and frequency to deduce an appropriate conduction mechanism and investigate different conduction parameters. According to the literature, no integrated investigation is present for the entire studied ferrites using egg white as a fuel in the autocombustion process.

## 2 Results and Discussion

**Figure 1:**
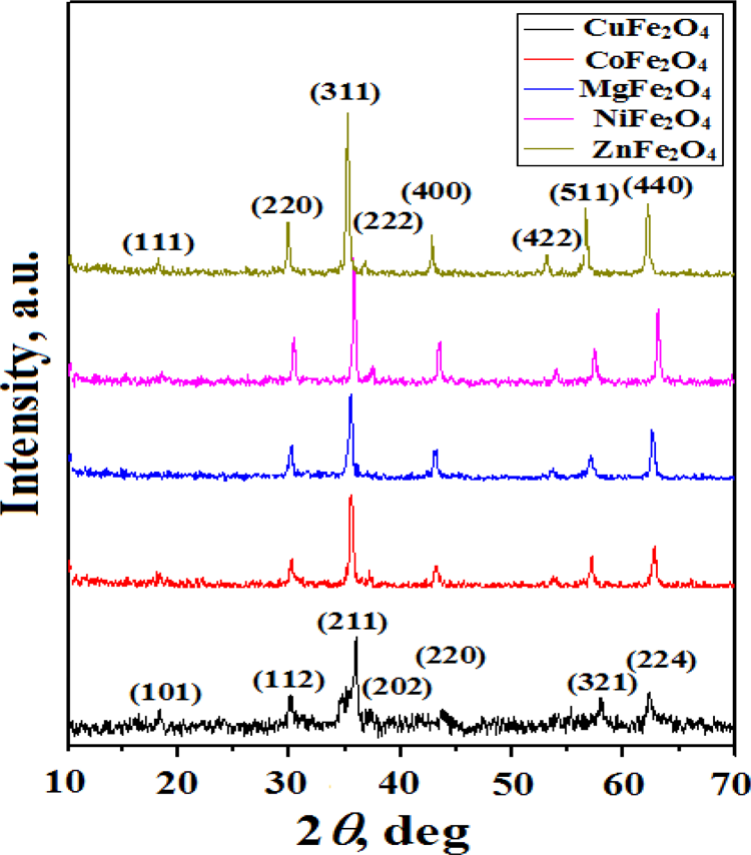
XRD patterns of the different as-prepared ferrites.

Diffraction patterns of the entire as-prepared ferrite samples (Figure 1) agreeing with the JCPDS card nos. 79-1744, 86-2267, 34-0425, 71-1232, and 22-1012 attributed to CoFe_2_O_4_, NiFe_2_O_4_, CuFe_2_O_4_, MgFe_2_O_4_, and ZnFe_2_O_4_ spinel ferrite phases, respectively, without any lines characteristic for any secondary phases. The obtained diffraction planes that appeared at (111), (220), (311), (222), (400), (422), (511), and (440) indicated cubic spinel phase formation for all the investigated samples. An exception was observed for the CuFe_2_O_4_ sample, indicating diffraction planes at (101), (112), (211), (202), (220), (321), and (224) characteristic for the body-centered tetragonal-phase structure. These results confirmed the effectiveness of the entire egg-white autocombustion method in preparing ferrite spinels in a short time without the need for any further heat treatments.

The lattice parameters (*a*) were calculated following the well-known Bragg equation by using interlayer spacing values (*d*) and reflections (*hkl*)^27^
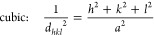
1
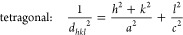
2

The calculated lattice parameters are summarized in Table 1. The obtained lower values compared to others prepared via aloe vera plant extract^18^ or coprecipitation^20^ routes may be due to the difference in the cation distribution inside crystal sublattices. X-ray density (*D*_x_) was estimated using the equation^27^
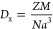
3where *Z* is the number of the molecules per unit cell, *M* = molecular weight, and *N* = Avogadro’s number. The reported values of X-ray density (Table 1) indicated an obvious lower value of density calculated for MgFe_2_O_4_ than the other ferrites, attributed to its lower molecular weight. The clear broadening of peaks (Figure 1) indicated the nanocrystalline nature of the entire ferrites. The average crystallite size (*L*) can be calculated by using Scherrer’s equation^27^
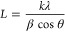
4where *k* is the correction factor = 0.94 that accounted for the particle shapes, β = full width at half-maximum of the peak, λ = wavelength (1.5406 Å), and θ is the Bragg angle. The reported values in Table 1 indicate a larger crystallite size with NiFe_2_O_4_ (47 nm) and the lower one with MgFe_2_O_4_ (27 nm). Generally, the obtained crystallite sizes showed smaller values than those estimated by Huang et al.^24^ for similar ferrites prepared via the citrate sol–gel method.

**Table 1:** Structural and Electromagnetic Data of the Studied Ferrites

ferrite	a (nm)	L (nm)	*D*_x_ (g cm^–3^)	υ _1_ (cm^–1^)	υ _2_ (cm^–1^)	D (nm)	*M*_S_ (emu/g)	*M*_r_ (emu/g)	*H*_C_ (Oe)	σ (Ω^–1^ cm^–1^)	ε′	ε″
CoFe_2_O_4_	8.3861	42	5.28	590	393	47	42.8	18.9	1667	2.3 × 10^–6^	31	41
NiFe_2_O_4_	8.3373	40	5.37	615	407	35	30.3	7.0	219	3.7 × 10^–6^	50	66
CuFe_2_O_4_	*a* = 5.8237, *c* = 8.7670	46	5.34	598	420	52	17.5	7.6	535	2.4 × 10^–6^	34	44
MgFe_2_O_4_	8.3651	27	4.54	577	432	30	12.9	2.5	184	1.9 × 10^–6^	32	36
ZnFe_2_O_4_	8.4299	42	5.35	550	400	48	2.1	0.2	170	8.3 × 10^–6^	97	149

**Figure 2:**
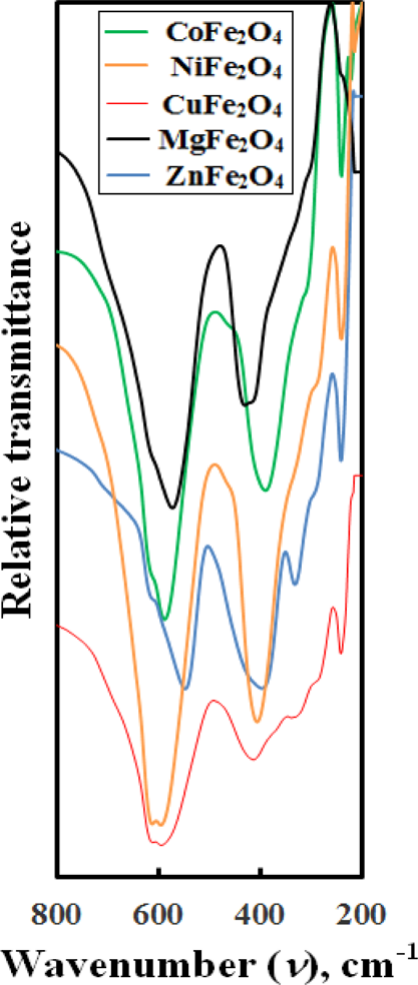
FT-IR spectral bands of the different as-prepared ferrites.

Infrared 1 spectra (Figure 2) clearly show two absorption bands between 350 and 650 cm^–1^ attributed to the oxygen ion vibration with tetrahedral and octahedral cations. According to Waldron,^28^ the high-frequency band, with υ_1_, corresponded to the metal–oxygen vibration at the tetrahedral sites. The low-frequency one, υ _2_, is due to the metal–oxygen vibrations at the octahedral (B) sites. The different obtained values of vibrational frequencies could be assigned to the bond length difference at different lattice sites. Generally, the bond length of tetrahedral is lower than that of the octahedral site.

The values of the vibrational frequencies (υ _1_ and υ _2_) are given in Table 1. We realize that the tetrahedral vibrational frequency, υ _1_, indicated obvious decrease in the order, NiFe_2_O_4_ → CuFe_2_O_4_ → CoFe_2_O_4_ → MgFe_2_O_4_ → ZnFe_2_O_4_, while the octahedral vibrational frequency, υ _2_, decreases in the order, MgFe_2_O_4_ → CuFe_2_O_4_ → NiFe_2_O_4_ → ZnFe_2_O_4_ → CoFe_2_O_4_. Generally, the frequency variation depends on the bond length of sublattice sites, cation distribution, and the mass number of cations preferentially occupying these sites in the spinel structure.^10,15,27^ The above orders could be discussed with consideration of ionic radii of different cations in both sites calculated according to Shannon tables^29^ in tetrahedral sites: *r*_Co^2+^_ = 0.58, *r*_Ni^2+^_ = 0.55, *r*_Cu^2+^_ = 0.57, *r*_Mg^2+^_ = 0.57, *r*_Zn^2+^_ = 0.60 and *r*_Fe^3+^_ = 0.49 Å and in octahedral sites: *r*_Co^2+^_ = 0.745, *r*_Ni^2+^_ = 0.69, *r*_Cu^2+^_ = 0.73, *r*_Mg^2+^_ = 0.72 *r*_Zn^2+^_ = 0.74 and *r*_Fe^3+^_ = 0.645 Å] taking their distribution preference in both sites^1^ and their atomic weights into consideration. A similar study was carried out by Prasad et al.^23^ for spinel ferrites prepared via a citrate sol–gel autocombustion route.

**Figure 3:**
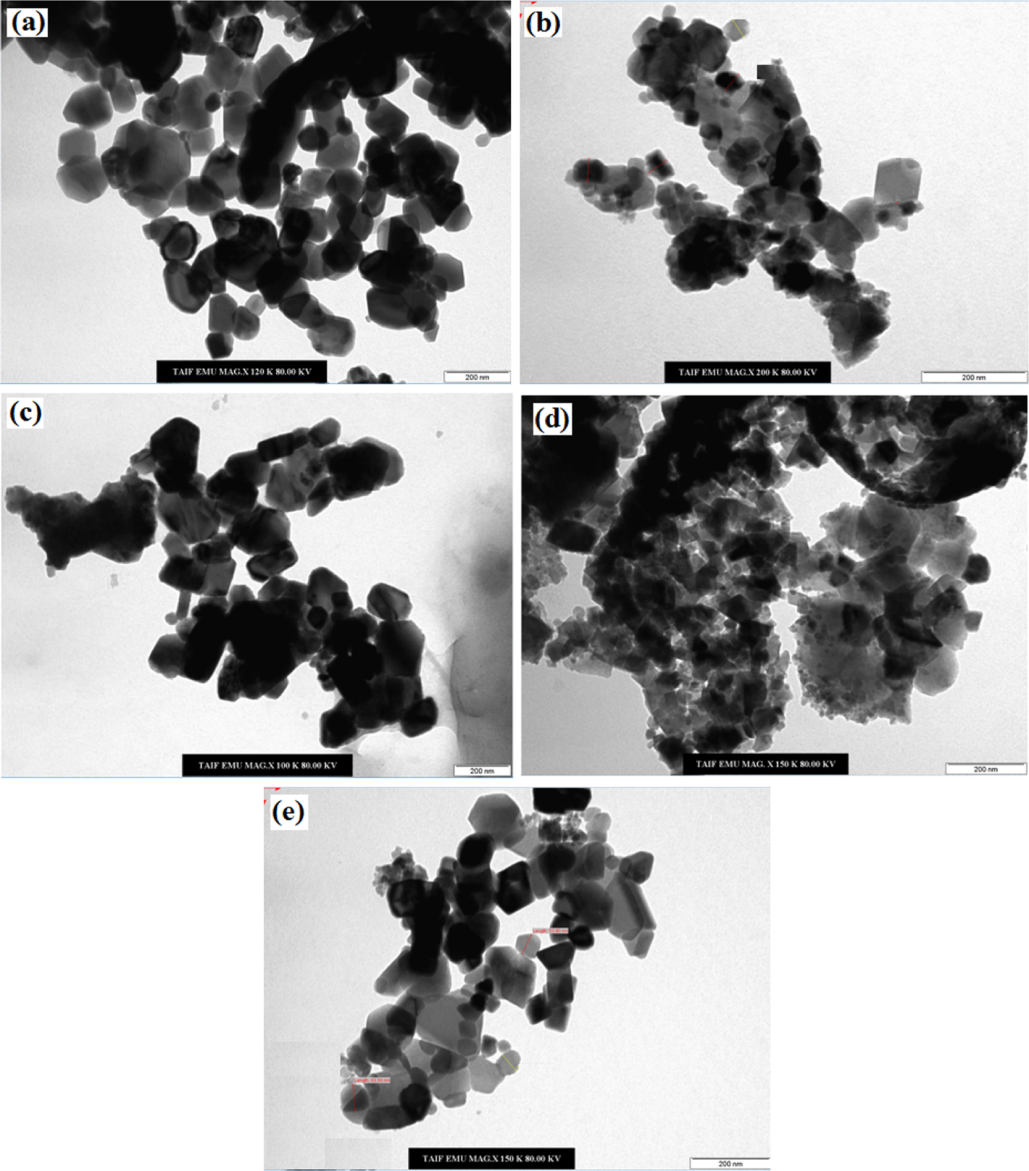
TEM images of the as-prepared different ferrites. (a) CoFe_2_O_4_, (b) NiFe_2_O_4_, (c) CuFe_2_O_4_, (d) MgFe_2_O_4_, and (e) ZnFe_2_O_4_. Scale bar = 200 nm.

Figure 3 exhibits TEM images of the entire prepared ferrites. The relatively large grains sizes (*D*) estimated (Table 1) compared to the crystallite sizes calculated via Scherrer’s equation are attributed to the agglomeration of the noticeable particles, considered as the normal phenomena of most studied magnetic nanoparticles.^13,15,27^ The images also indicate cubic morphology for all the studied ferrites except for the CuFe_2_O_4_ image (Figure 3c), which showed tetragonal morphology, in agreement with the XRD measurement results.

Phumying et al.^18^ reported similar agglomeration behavior using the TEM technique for ferrites prepared via the hydrothermal method using aloe vera extract. They showed that the prepared NiFe_2_O_4_, MgFe_2_O_4_, and CoFe_2_O_4_ samples contain nanoparticles, whereas the ZnFe_2_O_4_ samples contain a plate-like structure of a network of nanocrystalline particles. On the other hand, Deng et al.^10^ reported monodispersed microspheres for NiFe_2_O_4_, MgFe_2_O_4_, and CuFe_2_O_4_ prepared using the solvothermal method.

**Figure 4:**
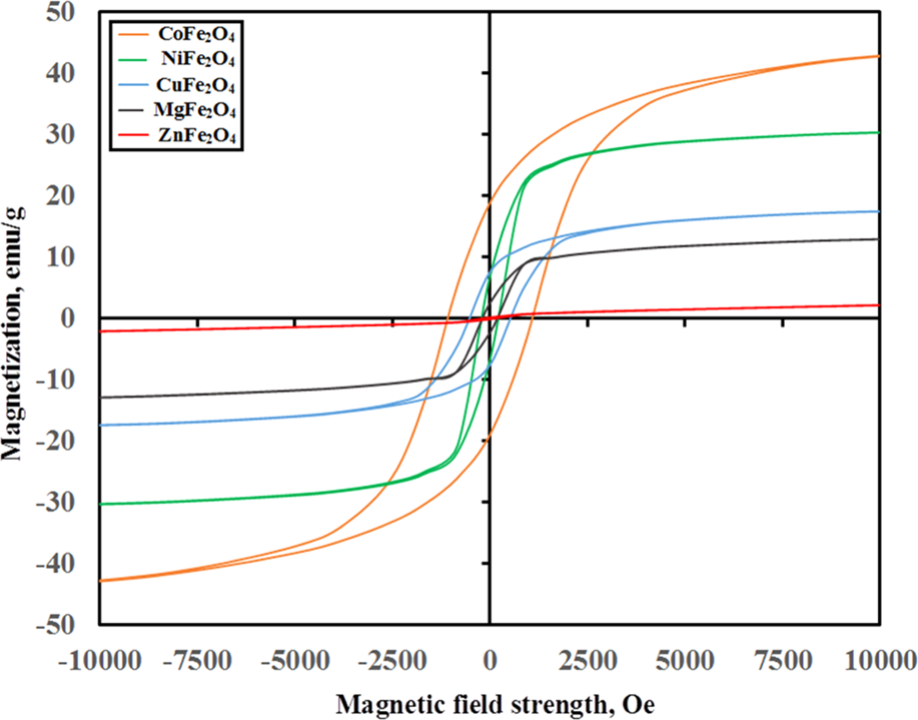
Hysteresis loops for the as-prepared different ferrites.

Room-temperature field-dependent magnetization for synthesized ferrite nanoparticles was measured up to 10 kOe. VSM hysteresis loops for all the prepared ferrites (Figure 4) indicated ferromagnetic characteristics and relatively low magnetization. The magnetic parameters, viz., saturation magnetization (*M*_s_), magnetic moment (η_B_), coercivity (*H*_c_), and remanent magnetization (*M*_r_), are evaluated in Table 1. The magnetization values were found to vary with the type of the metal ion, and CoFe_2_O_4_ showed the highest one (42.8 emu/g). This value was observed to be lowered to 30.3, 17.5, 12.9, and 2.1 for NiFe_2_O_4_, CuFe_2_O_4_, MgFe_2_O_4_, and ZnFe_2_O_4_, respectively. The magnetization value for CoFe_2_O_4_ was found to be lower than that of the bulk one (80 emu/g)^30^ and those prepared via other wet methods.^18,19,24,31−33^ On the other hand, the magnetization values obtained for the other ferrites are also lower than those reported in the literature.^18,19,24^ Generally, this reduction in the magnetization may be attributed to the smaller sizes of the prepared ferrites in comparison with those obtained by other methods, cation redistribution, or may be due to the presence of antiferromagnetic or magnetically dead layers on the ferrite’s surface.^34^ The obtained coercive field values (*H*_c_) showed an oscillation ranging from 1667 for CoFe_2_O_4_ to 170 emu/g for ZnFe_2_O_4_. The high coercivity values exhibited by CoFe_2_O_4_ and CuFe_2_O_4_ justified the high anisotropy and indicated a hard ferrite type with a high demagnetization field. This result suggests their use in magnetic recording media or permanent magnets. The obtained coercivity values of other ferrites indicated properties between hard and soft, so it is easy to change their magnetization, suggesting their use as magnetic field conductors. Generally, the obtained coercivity values are higher than their corresponding values reported in the literature,^18,19,24,32−34^ exhibiting the impact of the present synthesis route.

**Figure 5:**
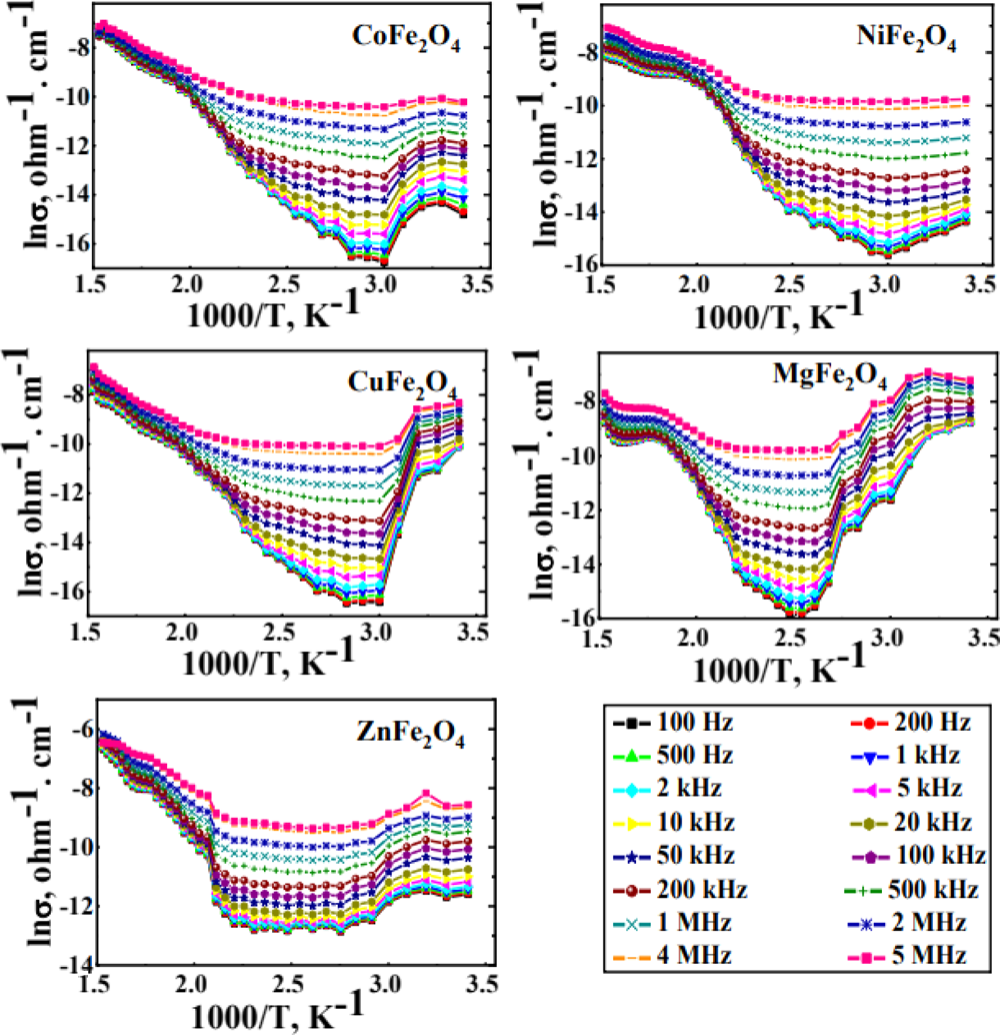
ln σ vs 1000/*T* as a function of frequency for the as-prepared different ferrites.

Figure 5 illustrates the ac-conductivity dependences on temperature as a function of frequency for the studied ferrites. The figure exhibited semiconducting behavior with three well-defined changes in the slopes of ln σ vs 1000/*T*. The first one showed a descending behavior with increasing temperature, while the others indicated an ascending behavior. The descending one could be assigned, according to the previous studies on similar ferrites,^31,35,36^ to surface-adsorbed water evaporation (formed during the pellets preparation for conductivity measurements). This type of water acts as the electron donor, and its evaporation thus decreases conductivity.

The slope change in the ascending part could indicate the change in the entire conduction mechanism. It was reported^37^ that the conduction in ferrites takes place through electron hopping between different valence ions of the same element. The iron content mainly provides this through the hopping of electrons between Fe^2+^ and Fe^3+^ ions present in the octahedral (B) sites. By increasing the temperature, the increased thermal energy promotes more electrons, thus increasing conductivity. A further elevation in the thermal energy increases lattice vibration and thus scatters the promoted electrons and changes the conduction into polaron-type one. CoFe_2_O_4_ prepared by different combustion routes reported similar behavior.^35^

**Figure 6:**
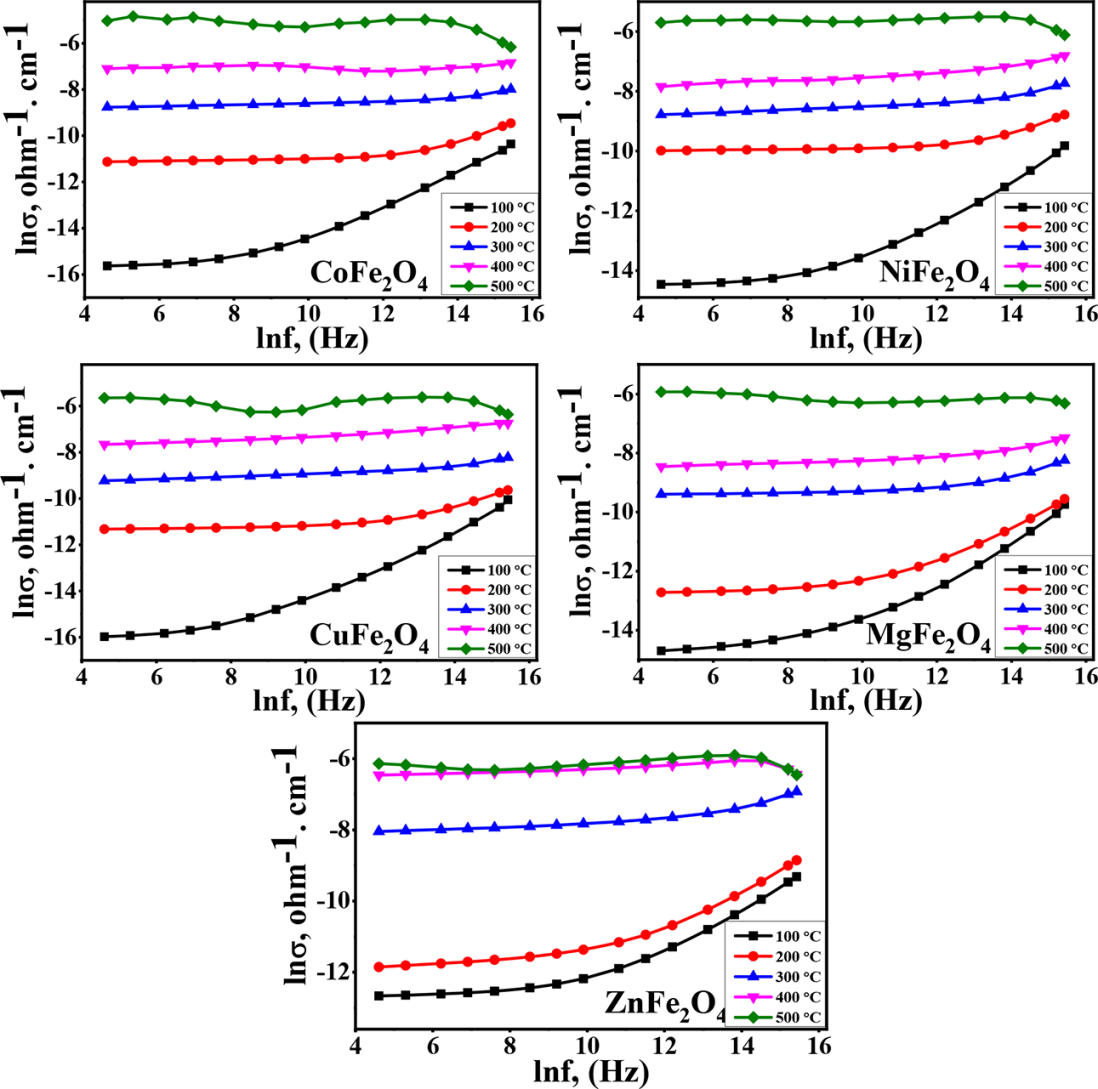
ln σ vs frequency as a function of temperatures for the different as-prepared ferrites.

This change in the entire conduction mechanism could be confirmed from the ac-conductivity versus frequency plot (Figure 6). The obvious gradual increase in conductivities at low temperatures followed by an almost linear trend could illustrate this change.^35^Table 1 summarizes the ac-conductivity of different ferrite samples measured at 400 K and 100 kHz. The maximum value was obtained for ZnFe_2_O_4_, while the lowest one was registered for MgFe_2_O_4_. This obtained variation in the conductivity may be due to the difference in ferrites’ cation distribution, affecting iron ions’ amount at octahedral sites.^20^ The values of ac-conductivities measured by Kurtan et al.^38^ for CoFe_2_O_4_ and ZnFe_2_O_4_ prepared via the oleyl amine route (2.54 × 10^–8^ and 2.56 × 10^–8^ Ω^–1^ cm^–1^, respectively) are about 100 times lower than the present obtained data, indicating the impact of the present egg-white route in improving conductivity.

In Figure 6, the conductivity measured at 100 K of all ferrites is observed to increase about 1000 times by increasing the temperature to 400 K at the same frequency, which showed the effect of the temperature on hopping conduction of these obtained semiconducting materials. For example, for CoFe_2_O_4_, the conductivity increases from 1.1 × 10^–7^ to 3.4 × 10^–4^ by the increasing temperature from 100 to 400 K at 10 kHz.

**Figure 7:**
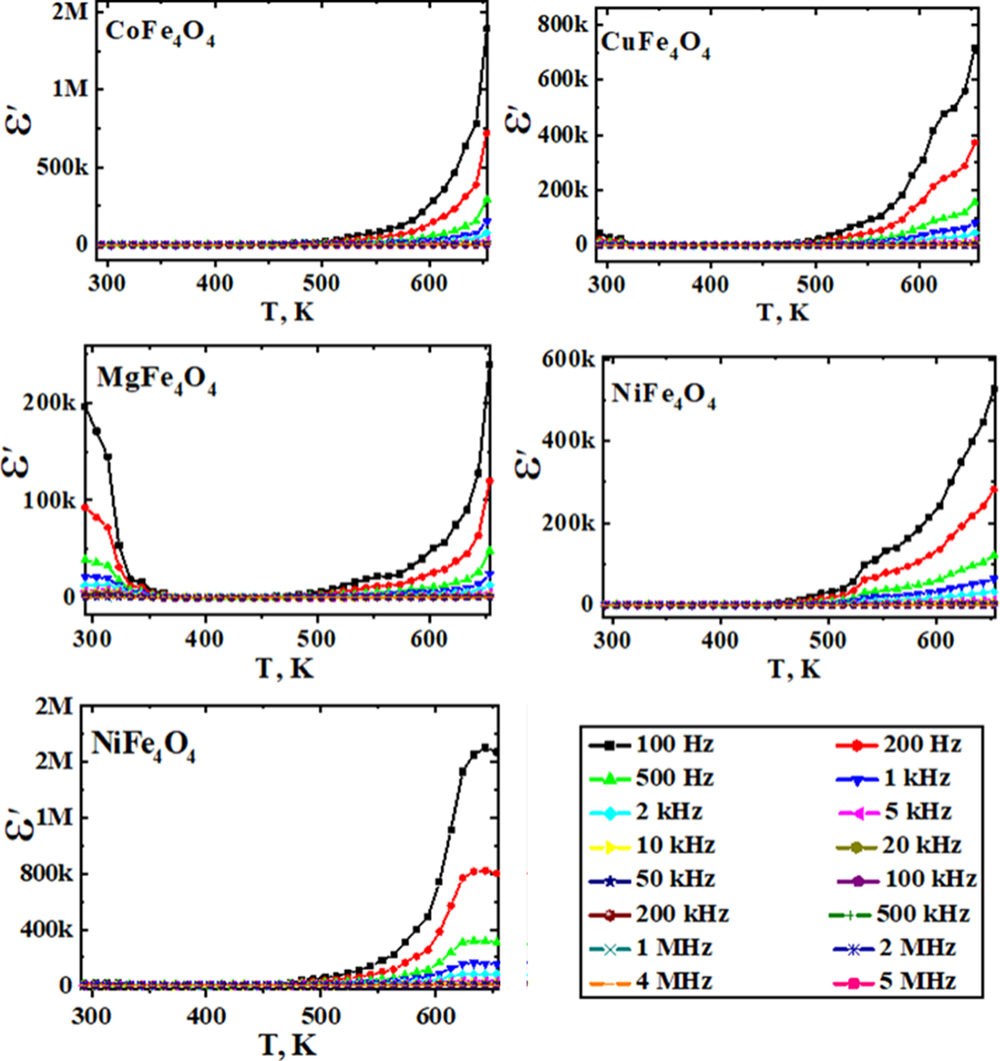
Dielectric constant (ε′) vs temperature as a function of frequency for the different as-prepared ferrites.

**Figure 8:**
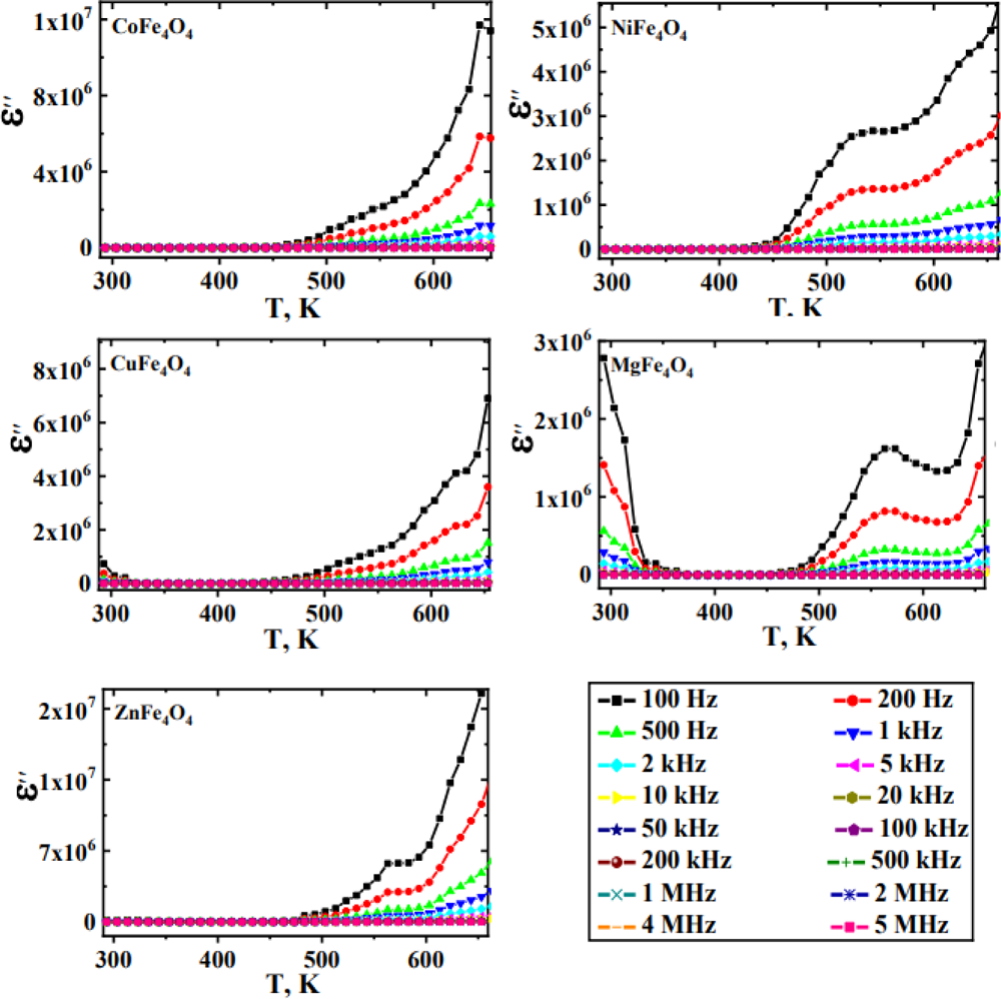
Dielectric constant (ε″) vs frequency as a function of temperatures for the different as-prepared ferrites.

The temperature dependences as a function of frequencies of real (ε′) and imaginary (ε″) dielectric constants for different studied ferrites are presented in Figures 7 and 8. Generally, dielectric constant values showed temperature and frequency independence up to about 500 K. At higher temperatures, the frequency and thermal energy effects predominate with a noticeable increase in dielectric constant values. At high temperatures, the increased thermal energy promotes hopping and polarization.^38^ Additionally, the observed decrease in the dielectric properties with the frequency increase indicates the inability of electrons to follow applied alternative electrical current beyond certain frequencies.^20,39^

Table 1 reports the dielectric constant values of different ferrites measured at 400 K and 100 kHz. The obtained results revealed, in agreement with conductivity measurements, higher values for ZnFe_2_O_4_ and lower ones with MgFe_2_O_4_. Also, the obvious relaxation appeared around 550 K for all studied ferrites (Figures 7 and 8), agreeing well with the observed change in the slope of conductivity versus reciprocal temperature (Figure 5). This result indicates occurrence of polarization in ferrites via a mechanism similar to that of the conduction process.^20^ The obtained low dielectric constant values could be enhanced using these ferrites in high-frequency applications such as microwave ones.

## 3 Conclusions

The present egg-white method indicated better promising results in preparing the entire ferrites over other conventional ceramic and wet chemical methods due to its simplicity, fast, economical, and environment friendly nature. The observed variations in the estimated properties of similar ferrites prepared by other methods were due to the cation redistribution between different lattice sites. The observed decrease in the magnetization value than ferrites prepared by other methods was attributed to the smaller particle sizes or the presence of antiferromagnetic or magnetically dead layers on the ferrite’s surface. The electrical conductivity and dielectric constant showed low values, suggesting using these ferrites in high-frequency applications such as microwaves.

## 4 Experimental Details

### 4.1 Materials

All used nitrates: Co(NO_3_)_2_·6H_2_O, Ni(NO_3_)_2_·6H_2_O, Fe(NO_3_)_3_·9H_2_O, Cu(NO_3_)_2_·6H_2_O, Mg(NO_3_)_2_·6H_2_O, and Zn(NO_3_)_2_·6H_2_O (BDH) were used as obtained. An aqueous egg-white solution was prepared by dissolving 60 g of fresh extract in distilled water under vigorous stirring for 30 min.

### 4.2 Procedure

A group of spinel ferrites (MFe_2_O_4_), M = Co, Ni, Cu, Mg, and Zn was synthesized via the autocombustion route using metal nitrates and egg-white fuel. The preparation details are reported in previous works.^13,15^ In brief, in an experiment for preparation of CoFe_2_O_4_, a stoichiometric amount of metal nitrates (Co/Fe = 1:2), equivalent to the formation of CoFe_2_O_4_ after autocombustion, was dissolved in 100 mL of distilled water. The egg-white aqueous solution was prepared by thoroughly mixing 60 ml of freshly extracted egg-white with 40 mL of distilled water. The nitrate solution was then added slowly under vigorous stirring to the egg-white solution. After 30 min of stirring, the formed gel was aged at about 80 °C until a dry gel precursor formed, which autocombusted by further heating on the hot plate with the evolution of dense gases. The obtained loose powder after complete combustion was given the name—the as-prepared precursor. The detailed mechanism for the ferrite formation using this route is already discussed elsewhere.^10^

### 4.3 Choice of Materials

Two main components must be present in all autocombustion reactions: the oxidant and the fuel between them; a redox reaction could occur. In the present study, the nitrate ion can act as an oxidant using egg-white as the fuel. The egg-white contains a mixture of numerous proteins formed by joining amino acids via a peptide bond. Thus, it could be considered as a natural-cum-gelling material, which thus acts as a binder for the entire metals. As soon as the redox reaction initiated, the accompanying energy released accelerated the autocombustion reaction, resulting in the ferrite production.^10^

### 4.4 Characterization

Single-phase formation was analyzed by XRD (D8 Advanced Diffractometer, Bruker AXS). The FT-IR spectra were measured using the KBr technique by a Jasco FTIR-310 spectrophotometer. The morphology was monitored using TEM (JEOL-2010) at 100 kV. The magnetic properties at room temperature were measured using a vibrating sample magnetometer (VSM-9600 M) up to 10 kOe. The electrical properties (ac-conductivity and dielectric constant) were measured using silver-coated pellets in the frequency range from 100 Hz to 5 MHz up to the temperature of 650 K by a Hioki 3531-LCR bridge.
